# Why Do Plants Convert Sitosterol to Stigmasterol?

**DOI:** 10.3389/fpls.2019.00354

**Published:** 2019-03-28

**Authors:** Siddique I. Aboobucker, Walter P. Suza

**Affiliations:** Department of Agronomy, Iowa State University, Ames, IA, United States

**Keywords:** stigmasterol, sterol end products, cell signaling, physiology, abiotic, sterol pathway regulation

## Abstract

A direct role for cholesterol signaling in mammals is clearly established; yet, the direct role in signaling for a plant sterol or sterol precursor is unclear. Fluctuations in sitosterol and stigmasterol levels during development and stress conditions suggest their involvement in signaling activities essential for plant development and stress compensation. Stigmasterol may be involved in gravitropism and tolerance to abiotic stress. The isolation of stigmasterol biosynthesis mutants offers a promising tool to test the function of sterol end products in signaling responses to developmental and environmental cues.

## Introduction

**Figure 1:**
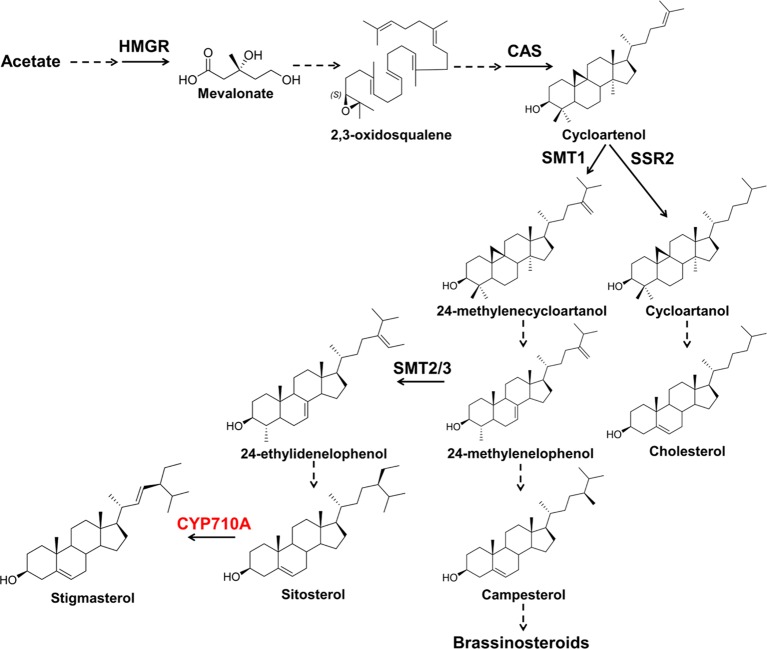
The plant sterol pathway leading to stigmasterol. Plants produce a mixture of sterols, campesterol (24-methyl) and sitosterol and stigmasterol (24-ethyl sterols). Stigmasterol is derived from sitosterol by the action of sterol C-22 desaturases. Campesterol is the preferred precursor of brassinosteroids (BR). Dashed arrows indicate multiple steps and solid arrows denote single step in the pathway. HMGR, 3-hydroxy-3-methylglutaryl-CoA reductase; CAS, cycloartenol synthase; SMT1, sterol methyltransferase 1; SMT2/3, sterol methyltransferase2/3; SSR2, sterol sidechain reductase2; CYP710A, sterol C-22 desaturase.

Unlike mammals and fungi, plants produce mixtures of sterols, including sitosterol, stigmasterol, campesterol, and cholesterol (Figure 1). The interaction of sterols with phospholipids helps plant cells to maintain plasma membrane fluidity and permeability during stress conditions (Grunwald, 1971; Hartmann, 1998). In addition, sterols are precursors in the synthesis of steroid hormones, e.g., testosterone, estrogen, glucocorticoids, and mineral corticoids in mammals, ecdysteroids in insects and crustaceans, antheridiol and oogoniol (mating hormones of fungi), and brassinosteroids (BR) in plants (Fujioka et al., 1997; Noguchi et al., 1999; Nomura et al., 1999; Clouse, 2002).

Campesterol is the precursor of BR (Fujioka and Yokota, 2003), and the crucial role of BR in plant growth and development is well established (Fujioka et al., 1997; Choe et al., 1998, 1999; Noguchi et al., 1999; Nomura et al., 1999; Clouse, 2002), while sitosterol is implicated in cellulose synthesis (Peng et al., 2002; Schrick et al., 2012). It is, however, unclear whether fluctuations in stigmasterol concentration observed during development and conditions of stress are responsible for cellular signaling. Nonetheless, evidence pointing to stigmasterol as a potential signal for cellular defense and gravitropic responses is emerging (Griebel and Zeier, 2010; Dalal et al., 2016). Functional characterization of genes controlling stigmasterol biosynthesis might help increase our understanding of the direct role of this sterol in plant development and stress responses. Therefore, our purpose is to examine genetic, developmental, and environmental conditions affecting stigmasterol production and suggest experimental approaches to investigate the role of stigmasterol in cell signaling.

## The Biosynthesis of Stigmasterol

Stigmasterol is produced in the mevalonate pathway following a series of enzyme-catalyzed reactions leading to the generation of 2,3-oxidosqualene (Schaller, 2003; Bach, 2016). Subsequently, 2,3-oxidosqualene is converted to cycloartenol by cycloartenol synthase (Schaller, 2003; Gas-Pascual et al., 2014; Sonawane et al., 2016). Cycloartenol is the target of branch-point enzymes including sterol side chain reductase 2 (SSR2) and sterol methyl transferase 1 (SMT1). SSR2 channels cycloartenol to the cholesterol branch, while SMT1 catalyzes the alkylation of cycloartenol to produce precursors for plant sterols (Benveniste, 1986; Nes and Venkatramesh, 1999; Diener et al., 2000; Schaeffer et al., 2001; Sonawane et al., 2016). Downstream of SMT1, other branching enzymes SMT2/3, directs carbon toward sitosterol and stigmasterol (Carland et al., 2010). Besides biosynthesis, free stigmasterol content can also be modulated by converting it to sterol conjugates such as steryl esters, steryl glucosides, and acyl steryl glucosides. Steryl esters are conjugated by acyl transferases (Chen et al., 2007; Bouvier-Navé et al., 2010) and the steryl glucosides by UDP-glucose: sterol glucosyltransferase (DeBolt et al., 2009).

Structurally, stigmasterol is similar to sitosterol but differs from sitosterol due to a double bond at position C-22 introduced by the sterol C-22 desaturase (Benveniste, 2002; Morikawa et al., 2006). *Arabidopsis* contains four genes encoding sterol C-22 desaturases belonging to the cytochrome P450, CYP710A superfamily (Benveniste, 2004; Morikawa et al., 2006) and two sterol C-22 desaturases are responsible for stigmasterol biosynthesis in *Physcomitrella patens*, a moss in which the major sterol is stigmasterol (Morikawa et al., 2009). The number of genes encoding/predicted to encode sterol C-22 desaturase, however, varies across species (Supplementary Table S1). It is noteworthy that SMT2/3 and CYP710A1 are the only two unique enzymes leading to the biosynthesis of stigmasterol in the stigmasterol branch, while both campesterol and stigmasterol branches (Figure 1) share intermediate enzymes. This is a case similar to cholesterol synthesis in plants, where the pathway involves both unique and shared enzymes with the campesterol branch (Sonawane et al., 2016).

## Developmental Regulation of Stigmasterol

In mammalian systems, cholesterol biosynthesis is regulated *via* negative feedback suppression of several key genes (Goldstein and Brown, 1990). Thus, analysis of gene transcripts may help increase our understanding of the regulation of sterol biosynthesis during plant development (Schrick et al., 2011; Sonawane et al., 2016; Suza and Chappell, 2016). For instance, in the developing seeds of tobacco (*N. tabacum*), pea (*Pisum sativum*), rape (*Brassica napus*), and seedlings of *N. benthamiana* and *B. napus*, increased gene expression and enzyme activities coincides with sterol accumulation (Harker et al., 2003; Schrick et al., 2011; Suza and Chappell, 2016). In addition, apical tissues of *B. campestris* contain high levels of cholesterol but exhibit a decline in cholesterol and a rise in sitosterol at later stages of development (Hobbs et al., 1996). Moreover, varying concentrations of stigmasterol and its precursor are noticeable at both the seed and whole plant developmental stages. For instance, during germination of tobacco seed, stigmasterol increases two-fold (Bush and Grunwald, 1972), and in mung bean (*Vigna radiata*) seedlings, younger sections of hypocotyls contain higher levels of stigmasterol compared to sitosterol (Stalleart and Geuns, 1994).

Stigmasterol content also increases in tomato (*Solanum lycopersicon*) during fruit ripening and is associated with an increase in *CYP710A11* gene expression (Whitaker and Gapper, 2008). In addition, in maize (*Zea mays*) seedlings, the concentration of stigmasterol is higher in roots than in shoots (Kemp et al., 1967). Similar to the findings of Kemp et al. (1967), *N. benthamiana* seedlings display striking differences in sterol composition between organs, with higher stigmasterol content in roots than in leaves (Suza and Chappell, 2016). In contrast, stigmasterol concentration is elevated in *P. sativum* leaves but lower in seeds (Schrick et al., 2011). Taken together, the developmental profile of sterols and gene expression data from *Arabidopsis* (Supplementary Figure S1) suggests highly coordinated regulation of stigmasterol metabolism in plants.

## Impact of Biotic and Abiotic Stress on Stigmasterol

In Solanaceous plants, e.g., potato (*Solanum tuberosum*), cholesterol production rises to match the demand for the synthesis of steroid glycoalkaloids in response to wounding or pathogen infection (Choi et al., 1992; Hartmann, 1998; Arnqvist et al., 2003). Similarly, pathogenic bacteria and reactive oxygen species stimulate the biosynthesis of stigmasterol in *Arabidopsis* (Griebel and Zeier, 2010; Sewelam et al., 2014). Furthermore, genes encoding sterol C-22 desaturase are responsive to phytohormones, suggesting a role for stigmasterol in various stress responses (Supplementary Figure S1). Indeed, the overexpression of one of the *Arabidopsis* stigmasterol biosynthesis genes resulted in enhanced resistance to bacterial pathogens (Wang et al., 2012). Recently, Gamir et al. (2017) reported that PATHOGENESIS-RELATED PROTEIN 1 (PR-1) can bind sterols including stigmasterol *in vitro*. The authors conclude that PR-1 inhibits pathogen growth by sequestering sterols from pathogens (Gamir et al., 2017). However, it remains to be demonstrated whether the predicted ability of PR-1 to bind stigmasterol has a real biological significance.

Stigmasterol concentration increases in roots of wheat (*Triticum aestivum*) exposed to salt (Magdy et al., 1994). In addition, salt-induced increase in stigmasterol is associated with salt exclusion capacity of citrus (*Citrus medica*) rootstocks (Douglas and Walker, 1983), possibly due to the activation of the plasma membrane H^+^-ATPase by stigmasterol (Grandmougin-Ferjani et al., 1997). The plasma membrane H^+^-ATPase is the primary transporter of protons out of the cell (Muramatsu et al., 2002), and its activity is essential for maintaining ion homeostasis (Niu et al., 1995). Indeed, stigmasterol treatment of germinating seeds improved salt tolerances of faba beans (*Vicia faba* L.) and flax (*Linum usitatissimum*) (Hashem et al., 2011; Hassanein et al., 2012).

Plants grown in saline conditions experience retardation of root growth, but Ca^2+^ supply ameliorates these deleterious effects of salinity stress (Shabala et al., 2003). The beneficial effect of Ca^2+^ in the context of salinity stress is associated with the stabilization of plasma membrane and enhanced exchange of cations such as Na^+^ (Hirschi, 2004). It appears that Ca^2+^ may stimulate stigmasterol production in roots (Pilar et al., 1993; Magdy et al., 1994), and possibly, the stigmasterol induced by Ca^2+^ affects the plasma membrane H^+^-ATPase (Grandmougin-Ferjani et al., 1997), leading to enhanced extrusion of Na^+^ from the cell (Qiu et al., 2003).

Stigmasterol is elevated at the expense of sitosterol in tomato (*Lycopersicon esculentum*) when stored at 15°C (Whitaker, 1991). Indeed, analysis of *Arabidopsis* over-expressing *AtCYP710A1* and *Atcyp710a1* mutant lines suggests a role for stigmasterol in tolerance to unfavorable temperatures (Senthil-Kumar et al., 2013). Higher levels of sitosterol are detected in etiolated barley (*Hordeum vulgare*) tissues compared to stigmasterol, but the two sterols are detected in equal amounts in green tissues (Bush et al., 1971). Similar to etiolated barley, soybean plants grown under filtered sunlight conditions accumulate sitosterol, while stigmasterol levels decrease (Izzo and Navari-Izzo, 1981). The fluctuations in stigmasterol content in response to various environmental cues suggest that the conversion of sitosterol to stigmasterol may modulate plant response to environmental stimuli.

## Potential Role for Stigmasterol in Cell Signaling

Cholesterol modulates its own biosynthesis in mammalian cells *via* negative feedback (Marigo and Tabin, 1996; Edwards and Ericsson, 1999). Research in *Solanum* species suggested the existence of analogous cholesterol feedback mechanisms in plants (Bhatt and Bhatt, 1984); however, the idea that cholesterol modulates sterol biosynthesis in plants did not escape skepticism, since unlike mammals, plants synthesize an array of sterol end products (Hartmann, 1998). Production of several sterol end products presents a challenge in elucidating role of sterol end products in cell signaling in plants.

Analysis of *Arabidopsis* sterol biosynthesis mutants suggests that sterols play critical roles in plant development independent of BR (Lindsey et al., 2003) by influencing position-dependent cell fate during embryogenesis (Jang et al., 2000; Schrick et al., 2000; Clouse, 2002). For example, the *fackel* mutants lacking a functional sterol C-14 reductase display embryonic defects and dwarfism at the seedling stage and produce less BR, but exogenous BR fails to complement the mutant (Mayer et al., 1991; Jang et al., 2000; Schrick et al., 2000), whereas the loss of SMT1 function in *smt1/cph* plants results in the accumulation of cholesterol, defective embryo development, and increased sensitivity to Ca^2+^. Similar to *fackel*, the defective phenotype of *smt1/cph* plants cannot be rescued by exogenous BR (Diener et al., 2000).

The *SMT2/3 (COTYLEDON VASCULAR PATTERNING1—CVP1)* locus converts 24-methylene lophenol to 24-ethylidene lophenol (Carland et al., 2002). Consequently, *Arabidopsis* plants overexpressing *SMT2* accumulate sitosterol at the expense of campesterol and display reduced stature and growth (Schaller et al., 1998; Schaeffer et al., 2001). The *smt2/cvp1* plants exhibit aberrant alignment of vascular strands and misshapen vascular cells, reduced levels of sitosterol, and higher concentration of campesterol (Schaeffer et al., 2001; Carland et al., 2002); however, the aberrant phenotype of *AtSMT2* and *smt2/cvp1* plants is not associated with defective BR signaling (Schaller et al., 1998; Schaeffer et al., 2001; Carland et al., 2002).

Another classic *Arabidopsis* sterol mutant is *hydra,* with defective embryonic morphogenesis, seedling cell patterning, and root growth (Lindsey et al., 2003). *HYDRA1* and *HYDRA2/FACKEL* encode sterol isomerase and C-14 reductase, respectively (Souter et al., 2002). Similar to *fackel*, *hydra* mutants produce less campesterol, but BR application does not rescue their phenotypic defects. Interestingly, both *hydra1* and *hydra2/fackel* mutants produce high levels of stigmasterol compared to the wild type (Souter et al., 2002). Whether dysregulation of stigmasterol is the cause for the pleiotropic defects in the *hydra* mutants is unclear.

The compactness in the packing of plasma membrane (PM) lipid bilayer acyl chains—referred as membrane order (or liquid-ordered)—is influenced by sterol composition (Roche et al., 2008). The separation of liquid-ordered and liquid-disordered phases in the PM is observed *in vivo* in tobacco cells (Gerbeau-Pissot et al., 2014). In “raft hypothesis,” stress induction can lead to the formation of larger structures (proposed lipid rafts) from liquid-ordered nanodomains enriched in sterols and sphingolipids (Lingwood and Simons, 2010). The interaction of sterols with phospholipids to form lipid rafts in mammalian membrane systems is crucial for correct signaling and activity of intrinsic membrane proteins. Lipid rafts are associated with many plant proteins involved in redox regulation, hormone transport and signaling, and ion homeostasis (Willemsen et al., 2003; Borner et al., 2005; Lefebvre et al., 2007; Zauber et al., 2014). Examples of proteins associated with lipid rafts and sterols include GLABRA2 (GL2), SCRAMBLED (SCM), and PIN-FORMED (PIN). PIN proteins are involved in the transport of auxin to mediate polar cell growth and root gravitropism (Moore, 2002). GL2 is a phospholipid/sterol-binding transcription factor involved in the regulation of root hair development (Masucci et al., 1996), whereas SCM is a receptor for positional cues to modulate expression of GL2 and other cell fate regulators during root hair development (Grierson et al., 2014). Indeed, proteome analysis of *smt1/cph*, with an altered plasma membrane composition, revealed a compromised cell signaling (Zauber et al., 2014). Sterol depletion in the plasma membrane by cyclodextrin and filipin suggests the sensing of modifications of cell environment at the PM is sterol dependent in plants, which can lead to adaptive cell responses through regulated signaling processes (Roche et al., 2008; Bonneau et al., 2010). In tobacco cells, the proportion of ordered phases transiently increased during the early steps of the signaling triggered by cryptogein and flagellin, two elicitors of plant defense reactions (Gerbeau-Pissot et al., 2014).

The composition of free sterols and sterol conjugates influences the liquid-ordered phase formation (Grosjean et al., 2015). Stigmasterol by itself lacks the ability to increase membrane order, whereas sitosterol and campesterol increase the order. However, by interacting together with glycosylinositolphosphoceramide, the major sphingolipid in plant, stigmasterol can increase the membrane order, while the interaction with glucosylceramide decreased the order. Sitosterol by itself induces the production of many small domains, which increases in size together with the addition of free sterol-sphingolipid and free sterol-sterylglycoside/acylsterylglycoside combination (Grosjean et al., 2015). These findings suggest a role for specific sterol species to fine tune the membrane sterol composition, thereby regulating signaling events.

The *orc* mutation is allelic to *SMT1*, and analysis of the *smt1^orc^* plants revealed trace amounts of stigmasterol and aberrant localization of PIN2 and PIN3 (Willemsen et al., 2003). Therefore, regulated membrane sterol composition is important for correct positioning of proteins, such as PIN, and physiological responses such as root gravitropism (Men et al., 2008). The *hydra2/fackel* plants show an ectopic expression of GL2 in trichoblasts, resulting in a glabrous root phenotype possibly due to a compromised function of GL2 (Souter et al., 2002). There is a possibility that GL2 activity in *hydra/fackel* plants is diminished by a sterol molecule which causes a conformational change blocking DNA interaction with certain *trans*-factors (Schrick et al., 2004). Conversely, a sterol or its derivative may bind GL2 and tether it to the membrane in a manner similar to the way cholesterol tethers Hedgehog in vertebrate systems (Jeong and McMahon, 2002). Since stigmasterol plays a role in cell proliferation (Hartmann, 1998) and *hydra/fackel* plants accumulate high levels of stigmasterol (Lindsey et al., 2003), a dysregulated metabolism of stigmasterol may interfere with various cellular processes during development. Indeed, GL2 expression is dysregulated in the developing siliques of the *Arabidopsis acbp1* mutant with high stigmasterol (Lung et al., 2017). Perhaps, the aberrant SCM distribution in roots of the *ugt80B1* is due to deficiency in a stigmasterol conjugate (Pook et al., 2017), offering additional support for a role for stigmasterol in cell signaling.

## Stigmasterol Role in Modulating Cell Biology

Stigmasterol is one of the major sterols in plasma membranes of plant cells and plays a role in cell proliferation (Hartmann, 1998) and activation of plasma membrane H^+^-ATPase (Grandmougin-Ferjani et al., 1997). In plants, the plasma membrane H^+^-ATPase is the primary transporter of protons out of the cell, thus creating a pH and electrochemical gradient across the plasma membrane (Muramatsu et al., 2002). The activity of the plasma membrane H^+^-ATPase is essential for maintaining ion homeostasis, since carrier-mediated ion transport is coupled to a downhill pH gradient (Niu et al., 1995). In addition, the activity of the plasma membrane H^+^-ATPase promotes the adaptation of maize roots to low pH (Yan et al., 1998) and low phosphorous availability in soybeans (Shen et al., 2006).

In *Arabidopsis*, exogenous stigmasterol activates the expression of genes involved in cell expansion and division (He et al., 2003). Furthermore, exogenous stigmasterol increases flower numbers of chamomile (*Chamomilla recutita* L. Rausch) (Abd El-Wahed and Krima, 2004) and *in vitro* multiplication of shoots of Marubakaido apple rootstock (*Malus prunifolia* (Wild.) Borkh) (Pereira-Netto, 2012). In tobacco seeds, depletion of cycloartenol by increased activity of SMT1 was associated with elevated activity of HMGR in tobacco seeds (Holmberg et al., 2002). By contrast, studies in *P. sativum* showed that stigmasterol inhibits HMGR activity (Stermer et al., 1994), suggesting a role for stigmasterol in regulation of sterol biosynthesis.

In *Arabidopsis*, the key gene controlling stigmasterol production is *CYP710A1,* but *Arabidopsis* also produces low levels of brassicasterol from the C-22 desaturation of epi-campesterol by CYP710A2 (Benveniste, 2002; Morikawa et al., 2006). The expression of *CYP710A2* mRNA responds rapidly to gravity stimulation (Kimbrough et al., 2004), suggesting a role for sterol C-22 desaturation in plants response to gravity. The recent discovery that InteractoR Of SYnaptotagmin1 (ROSY1), a regulator of cellular trafficking and gravitropic response binds stigmasterol (Dalal et al., 2016), supports the idea that *CYP710A* genes and stigmasterol play a role in root response to gravity. In addition, the *rosy1-1* mutant is impaired in auxin transport but is more tolerant to salt stress (Dalal et al., 2016), suggesting a connection between ROSY1 and stigmasterol in regulating auxin transport and abiotic stress responses.

Stigmasterol is induced by Ca^2+^ (Pilar et al., 1993), and *Arabidopsis* mutants defective in Ca^2+^ uptake have a compromised cell expansion, short root hairs, and stunted roots (Foreman et al., 2003). Since gravistimulation induces Ca^2+^ (Monshausen et al., 2011), Ca^2+^ may stimulate *CYP710A2* expression and stigmasterol production in roots. Therefore, CYP710A proteins might participate in a similar signaling pathway with ROSY1 to modulate plant cell response to gravity and salt stress (Dalal et al., 2016).

## Concluding Remarks and Future Directions

**Figure 2:**
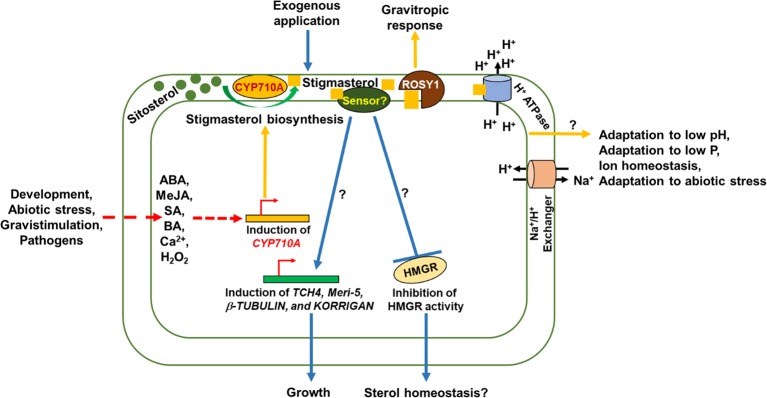
Schematic representation of functions of stigmasterol and its signaling roles in plant cells. Stigmasterol biosynthesis occurs during development, abiotic stress, gravistimulation, pathogen attack, and in response to signaling molecules such as abscisic acid (ABA), methyl jasmonate (MeJA), salicylic acid (SA), calcium (Ca^2+^), and hydrogen peroxide (H_2_O_2_). Stigmasterol binds to ROSY1 leading to gravitropic response. Stigmasterol activates H^+^-ATPase to create pH and electrochemical gradient across the plasma membrane. The pH gradient leads to activation of the Na^+^/H^+^ exchanger to exclude Na^+^ to adapt to salinity stress. Furthermore, the activity of H^+^-ATPase is necessary for ion homeostasis, adaptation to low pH, low phosphorus, and abiotic stress. Exogenous application of stigmasterol impacts growth and sterol homeostasis *via* an unidentified (stigma)sterol sensor.

There is need to validate gene expression data from microarray experiments (Supplementary Figure S1) and correlate hormone/stress induced gene expression with stigmasterol concentration. In addition, it is intriguing that blocking BR biosynthesis affects *CYP710A* gene expression (Supplementary Figure S1), suggesting a role for BR in regulating stigmasterol metabolism. Based on the mechanism for cholesterol and lipid sensing in mammals and insects, the notion of plant (stigma)sterol sensor(s) is not far-fetched. Indeed, the discovery of plant proteins with sterol/lipid sensing/binding domains offers a promising avenue for testing the signaling role of stigmasterol (Figure 2).

The potential candidate for a stigmasterol sensing system would be ROSY1, which shows binding specificity for stigmasterol to regulate root response to gravity (Dalal et al., 2016). Other candidates include *Arabidopsis* Niemann-Pick disease type C like proteins (AtNPC1-1 and AtNPC1-2) (Feldman et al., 2015), possessing putative sterol sensing domains reminiscent of SCAP and related regulators of sterol metabolism in animals and yeast (Goldstein and Brown, 1990; Nohturfft and Losick, 2002). Evaluating the sterol binding specificity of plant NPC proteins might provide clues as to whether AtNPC1-1 and AtNPC1-2 act as sterol sensors to modulate lipid metabolism; however, testing the implication of stigmasterol interaction with plant sterol sensing proteins requires circumventing gene redundancy (Supplementary Table S1). The creation of double/triple/quadruple mutants for *Arabidopsis CYP710A* genes may help in overcoming the challenge. Alternatively, crop or model grass species predicted to encode single copies of *CYP710A* (Supplementary Table S1), and rich genetic resources, such as maize or Brachypodium, may provide an opportunity to attempt to eliminate the production of stigmasterol *via* insertional mutagenesis or gene editing approaches.

Sterol glucosides are synthesized at the PM (Zauber et al., 2014), while the CYP710A is predicted to localize to the apoplast (Supplementary Table S1). This begs the question of what would be the cellular site of stigmasterol synthesis, since plant sterols are believed to originate primarily within the ER (Hartmann, 1998). Experiments to test the impact of ectopic expression of CYP710A via retention to ER or vacuole may help identify the preferred site of stigmasterol synthesis. This information will be helpful in designing gene constructs to manipulate stigmasterol content in a more targeted fashion.

## Data Availability

Publicly available datasets were analyzed in this study. These data can be found here: https://www.arabidopsis.org/servlets/Search?type=general&search_action=detail&method=1&show_obsolete=F&name=cyp710a&sub_type=gene&SEARCH_EXACT=4&SEARCH_CONTAINS=1.

## Author Contributions

WS and SA designed the research and wrote the paper.

### Conflict of Interest Statement

The authors declare that the research was conducted in the absence of any commercial or financial relationships that could be construed as a potential conflict of interest.

## Abbreviations

BR: Brassinosteroid
CPH: Cephalopod
CVP: Cotyledon vascular patterning
CYP710A: Sterol C-22 desaturase
GL2: GLABRA2
NPC: Niemann-Pick disease type C
PIN: PIN-FORMED
PR-1: PATHOGENESIS-RELATED PROTEIN 1
PM: Plasma membrane
ROSY1: InteractoR of SYnaptotagmin
SCM: SCRAMBLED
SMT: Sterol methyltransferase
SSR: Sterol sidechain reductase.
