# Supplementary material for: Why Do Plants Convert Sitosterol to Stigmasterol?
Source: Front Plant Sci. 2019 Mar 28;10:354. doi: 10.3389/fpls.2019.00354 (PMC6447690; doi:10.3389/fpls.2019.00354)
Supplement: Supplementary file 1 [file Data_Sheet_1.docx]

Supplementary Material

Why do plants convert sitosterol to stigmasterol?

Siddique I. Aboobucker, Walter P. Suza^*^

*** Correspondence:** Corresponding Author: [wpsuza@iastate.edu](mailto:wpsuza@iastate.edu)

# Supplementary Figures

**
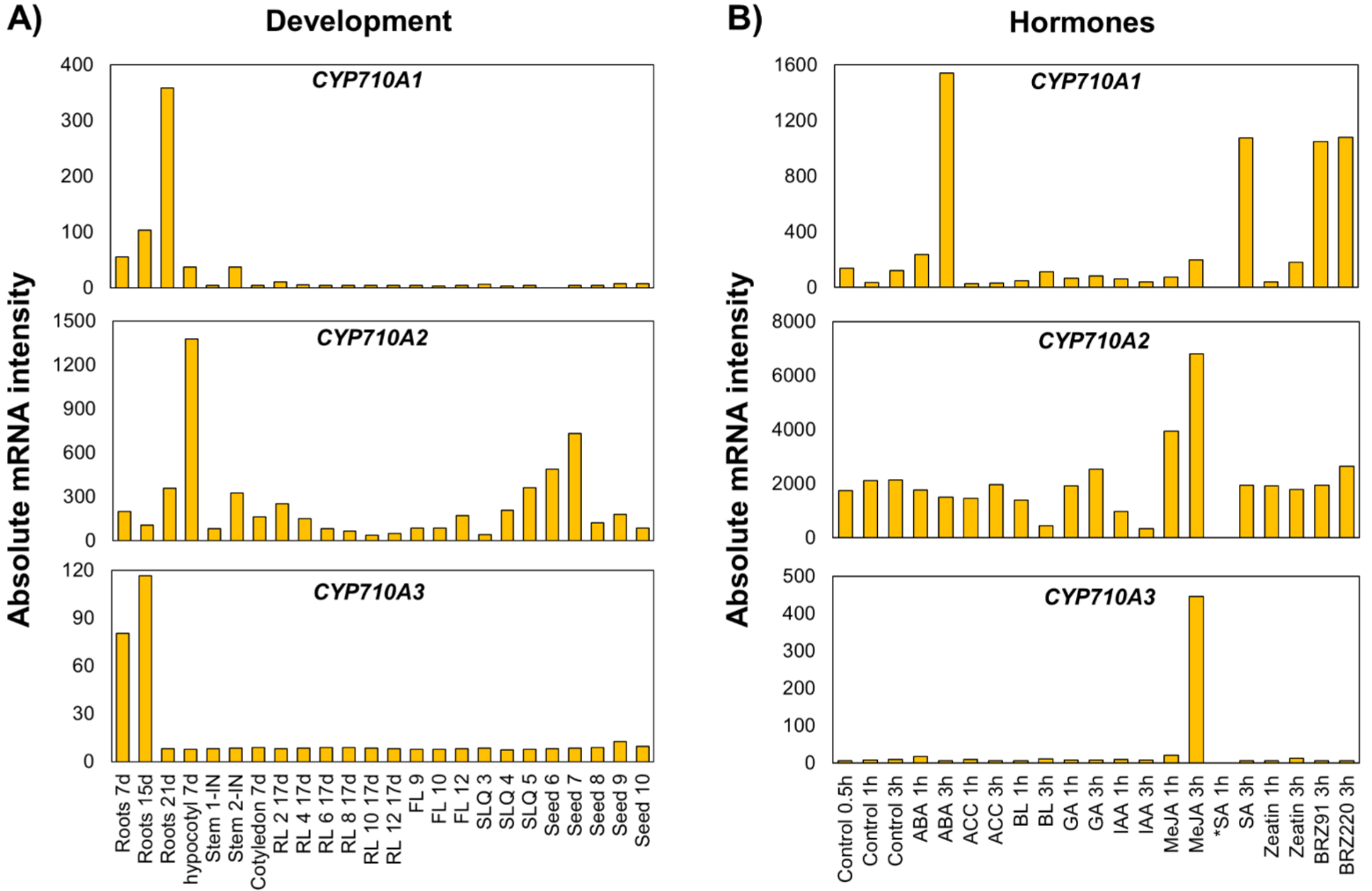


Supplementary Figure 1.** **(A)** Absolute mRNA intensity (background adjusted by gcRMA) of stigmasterol biosynthesis genes during various development stages of *Arabidopsis* WT Col-0. **(B)** Absolute mRNA intensity (background corrected by gcRMA) values of stigmasterol biosynthesis genes in *Arabidopsis* WT Col-0 seedlings treated with various hormones. Microarray ATH1 expression data in (A) and (B) were obtained from the AtGenExpress dataset (Schmid et al., 2005; Goda et al., 2008). The expression data for CYP710A4 is not available and therefore it is not included.

# Supplementary Tables

**Table S1.** Validated and predicted sterol C-22 desaturases in model and selected crop species

| **Group** | **Species** | **Copy Number** | **Gene Identifier(s)** | **Genomic structure** | **Protein Length** | **Subcellular Localization ^$^** |
| --- | --- | --- | --- | --- | --- | --- |
| Monocots | *Brachypodium distachyon* | 1 | Bradi_2g06770 | Intron less | 509 | Secretory pathway |
|  | *Oryza sativa* | 4 | Os01g11270, Os01g11280,  Os01g11300, Os01g11340 | Intron less | 504, 508, 511, 522 | Secretory pathway,  Other |
|  | *Sorghum bicolor* | 1 | Sb3g002060 | Intron less | 520 | Secretory pathway |
|  | *Triticum aestivum ^a^* | 3 | CYP710A8-A, CYP710A8-B, CYP710A8-D | Intron less | 510, 510, 511 | Secretory pathway |
|  | *Zea mays* | 1 | Zm00001d039384 | Intron less | 516 | Secretory pathway |
| Eudicots | *Arabidopsis thaliana ^b^* | 4 | AtCYP710A1, AtCYP710A2, AtCYP710A3, AtCYP710A4 | Intron less | 493, 493, 495, 499 | Secretory pathway  (Apoplast, PM) ^#^ |
|  | *Glycine max* | 2 | Glyma.13g217400, Glyma.15g095000 | Intron less | 513, 521 | Secretory pathway |
|  | *Lycopersicon esculentum ^b^* | 1 | Solyc02g070580 | Intron less | 501 | Secretory pathway |
|  | *Medicago truncatula* | 1 | Medtr2g019640 | Intron less | 515 | Secretory pathway |
|  | *Nicotiana tabacum* | 2 | LOC107763057, LOC107771426 | Intron less | 506, 506 | Secretory pathway |
|  | *Phaseolus vulgaris* | 1 | Phvul.006G163800 | Intron less | 626 | Other |
|  | *Solanum tuberosum* | 1 | Sotub02g016960 | Intron less | 501 | Secretory pathway |

^$^ Subcellular localization was predicted using TargetP (Emanuelsson et al., 2007). “Other” – not predicted to chloroplast, mitochondria or secretory pathway but to elsewhere.

^#^ Prediction consensus based on the SUBcellular location database for *Arabidopsis* proteins (SUBA) (Hooper et al., 2017)

^a^ (Tang et al., 2011); ^b^ Only AtCYP710A1, A2 and A4 have been experimentally validated (Arnqvist et al., 2008; Morikawa et al., 2006).

**References:**

Arnqvist, L., Persson, M., Jonsson, L., Dutta, P.C., and Sitbon, F. (2008). Overexpression of CYP710A1 and CYP710A4 in transgenic *Arabidopsis* plants increases the level of stigmasterol at the expense of sitosterol. *Planta* 227(2)**,** 309-317. doi: 10.1007/s00425-007-0618-8.

Emanuelsson, O., Brunak, S., von Heijne, G., and Nielsen, H. (2007). Locating proteins in the cell using TargetP, SignalP and related tools. *Nature Protocols* 2(4)**,** 953-971. doi: 10.1038/nprot.2007.131.

Goda, H., Sasaki, E., Akiyama, K., Maruyama-Nakashita, A., Nakabayashi, K., Li, W., et al. (2008). The AtGenExpress hormone and chemical treatment data set: experimental design, data evaluation, model data analysis and data access. *The Plant Journal* 55(3)**,** 526-542. doi: 10.1111/j.0960-7412.2008.03510.x.

Hooper, C.M., Castleden, I.R., Tanz, S.K., Aryamanesh, N., and Millar, A.H. (2017). SUBA4: the interactive data analysis centre for *Arabidopsis* subcellular protein locations. *Nucleic Acids Research* 45(D1)**,** D1064-D1074. doi: 10.1093/nar/gkw1041.

Morikawa, T., Mizutani, M., Aoki, N., Watanabe, B., Saga, H., Saito, S., et al. (2006). Cytochrome P450 CYP710A encodes the sterol C-22 desaturase in *Arabidopsis* and tomato. *The Plant Cell* 18(4)**,** 1008-1022. doi: 10.1105/tpc.105.037012.

Schmid, M., Davison, T.S., Henz, S.R., Pape, U.J., Demar, M., Vingron, M., et al. (2005). A gene expression map of *Arabidopsis thaliana* development. *Nature Genetics* 37(5)**,** 501-506. doi: 10.1038/ng1543.

Tang, J., Ohyama, K., Kawaura, K., Hashinokuchi, H., Kamiya, Y., Suzuki, M., et al. (2011). A new insight into application for barley chromosome addition lines of common wheat: achievement of stigmasterol accumulation. *Plant Physiology* 157(3)**,** 1555-1567. doi: 10.1104/pp.111.183533.
